# Patient-specific finite element analysis of orbital biomechanical responses to malpositioned self-inflating hydrogel implants in congenital microphthalmia

**DOI:** 10.3389/fmed.2026.1899892

**Published:** 2026-08-17

**Authors:** Dongyu Song, Qiguo Rong, Shen Yu, Lei Liu, Dongmei Li

**Affiliations:** 1 Department of Ophthalmology, Chaoyang Central Hospital, Chaoyang, Liaoning, China; 2 Department of Mechanics and Engineering Science, College of Engineering, Peking University, Beijing, China; 3 State Key Laboratory of Structural Analysis for Industrial Equipment, Dalian University of Technology, Dalian, Liaoning, China; 4 Guangdong Eye Institute, Department of Ophthalmology, Guangdong Provincial People’s Hospital (Guangdong Academy of Medical Sciences), Southern Medical University, Guangzhou, Guangdong, China; 5 Beijing Tongren Eye Center, Beijing Tongren Hospital, Capital Medical University, Beijing Ophthalmology and Visual Sciences Key Laboratory, Beijing, China

**Keywords:** biomechanics, congenital microphthalmia, finite element analysis, ophthalmology (MeSH), self-inflating spherical hydrogel

## Abstract

**Purpose:**

Congenital microphthalmia is characterized by arrested ocular development, resulting in restricted biomechanical stimulation associated with orbital bone growth and craniofacial deformity on the affected side. Self-inflating spherical hydrogel orbital implantation has been clinically used to compensate for orbital volume deficiency and stimulate orbital growth. However, postoperative hydrogel implant malposition occasionally occurs. This study aimed to investigate the biomechanical effects of displaced self-inflating spherical hydrogel implants on biomechanical stimulation associated with orbital bone growth using a three-dimensional finite element model of congenital microphthalmia.

**Methods:**

Orbital CT data from a child with congenital microphthalmia were selected from the institutional database. CT images were imported into a simulation platform to reconstruct the geometric structure of the skull. Based on CT measurements of the globe and extraocular muscles, three-dimensional CAD models of the globe, extraocular muscles, and self-inflating spherical hydrogel implant were generated. A three-dimensional finite element model of the orbital bones and intraorbital tissues was then established. A 3-mL self-inflating spherical hydrogel implant was placed at two extraconal retrobulbar positions: inferonasal (Model 1) and inferotemporal (Model 2). The expansion process of the hydrogel implant was simulated, and finite element analysis was performed to obtain stress distribution and radial displacement maps of the orbital bones for biomechanical evaluation.

**Results:**

Model 2 showed higher orbital biomechanical response than Model 1. Von Mises stress increased from 10.15–278.15 kPa (Model 1) vs. 200.05–425.36 kPa (Model 2) with hydrogel expansion (3–9×), and displacement reached 0.406 μm vs. 0.703 μm, respectively. Stress and displacement were concentrated near implant-adjacent walls, with inferotemporal dominance in Model 2. Central-point analysis confirmed consistent stress–displacement coupling, with greater overall and regional orbital deformation in Model 2.

**Conclusion:**

In children with congenital microphthalmia, displaced hydrogel implants located in the deep inferotemporal orbit may still effectively stimulate biomechanical stimulation associated with orbital bone growth and therefore may support careful clinical observation in selected cases and warrants further validation in larger clinical studies. However, when malposition occurs in the superficial inferotemporal orbit and causes visible cutaneous protrusion, surgical correction should be considered.

## Introduction

Congenital microphthalmia is a hereditary ocular maldevelopment characterized by arrested globe growth due to abnormal optic vesicle development during embryogenesis, with axial length typically less than 15 mm (1–5). Epidemiological data show that the prevalence of congenital microphthalmia in China is approximately 9 per 100,000, while in other countries it ranges from 8 to 32 per 100,000 (6–12). It accounts for 3.2–11.2% of inherited congenital blinding ocular diseases, ranking as the leading cause (12–14). There is no clear sex predilection, and approximately 43.9% of cases are unilateral (15). In unilateral cases, the absence of normal ocular stimulation leads to marked underdevelopment of the ipsilateral orbital bone and surrounding soft tissues, resulting in complex craniofacial deformities and significant facial asymmetry, which can severely impact psychological development and may lead to behavioral and personality disorders (16, 17).

The primary goal of management is to promote biomechanical stimulation associated with orbital bone growth and improve cosmetic appearance rather than visual restoration (15, 18). Currently, our ophthalmic center applies self-inflating spherical hydrogel orbital implantation to compensate for orbital volume deficiency and stimulate orbital development, achieving favorable clinical outcomes (19, 20). The hydrogel is composed of highly hydrophilic compounds, including N-vinylpyrrolidone and methyl methacrylate, and expands by osmotic water absorption from surrounding tissues, with its expansion volume and rate precisely predetermined during manufacturing. These spherical hydrogels are available in volumes ranging from 1 to 5 mL, allowing selection according to orbital cavity size (19). However, during intraorbital expansion within the muscular cone, some implants may displace outside the cone, most commonly into inferonasal or inferotemporal orbit due to gravity. The biomechanical differences of such malpositioned implants on orbital bone development, and whether surgical repositioning is necessary, remain unclear, as these effects cannot be directly measured in postoperative congenital microphthalmia patients.

Finite element analysis (FEA) is a well-established computational approach for evaluating biomechanical behavior in anatomically complex structures. By simulating stress distribution, deformation, and mechanical interactions under different loading conditions, FEA provides a non-invasive means of investigating tissue biomechanics when direct experimental measurement is impractical or impossible. This technique is particularly suitable for studying orbital biomechanics because of the complex geometry and heterogeneous mechanical properties of the orbital bones and intraorbital tissues. However, despite the advantages of FEA in biomechanical simulation, little is known about the mechanical consequences of postoperative malposition of self-inflating hydrogel implants used for orbital expansion in congenital microphthalmia. In particular, the biomechanical differences between inferonasal and inferotemporal implant malposition and their potential implications for the mechanical environment of the developing orbit have not been systematically investigated. This represents an important clinical knowledge gap.

Therefore, we developed a patient-specific three-dimensional finite element model to compare the orbital biomechanical responses associated with inferonasal and inferotemporal malposition of self-inflating hydrogel implants. By characterizing stress distribution and orbital wall displacement, this study aimed to provide biomechanical evidence that may facilitate postoperative assessment and generate hypotheses regarding implant positioning in congenital microphthalmia.

## Methods

This study was approved by the Ethics Committee of Chaoyang Central Hospital (No. CYSZXYY 2022-13) and adhered to the provisions of the Declaration of Helsinki for research involving human subjects.

### Three-dimensional reconstruction of the orbit

A 2-year-old boy who had been diagnosed with left eye blindness caused by congenital microphthalmia through clinical eye and brain computed tomography (CT) examinations was selected from the database of congenital microphthalmia patients in the eye center of Beijing Tongren Hospital as the subject of the study. Data use was authorized by the child’s guardian. The imaging data obtained from the database were CT scans of the head, using a Brilliance CT 64-channel scanner (Philips Medical Systems Inc., Cleveland, Ohio, USA), including 356 coronal section images, 360 median sagittal section images, and 256 transverse section images. Images were reconstructed on a 512 × 512 pixel matrix at 0.67-mm thickness. The ALARA (as low as reasonably achievable) principle was adhered to in the image generation process to minimize radiation exposure (10, 21, 22). The digital imaging and communications in medicine files of CT scans were transferred to the interactive medical image control system Mimics 17.0 (MIMICS, Materialise, Belgium) to reconstruct a geometrical skull and orbit. The eyeball and eye muscle geometries of the model were measured using CT data. Eyeball, eye muscle, and self-inflating tissue expanders were created with SolidWorks 2016 (SolidWorks, USA).

### Poroelastic finite element model of the orbit

**Figure 1:**
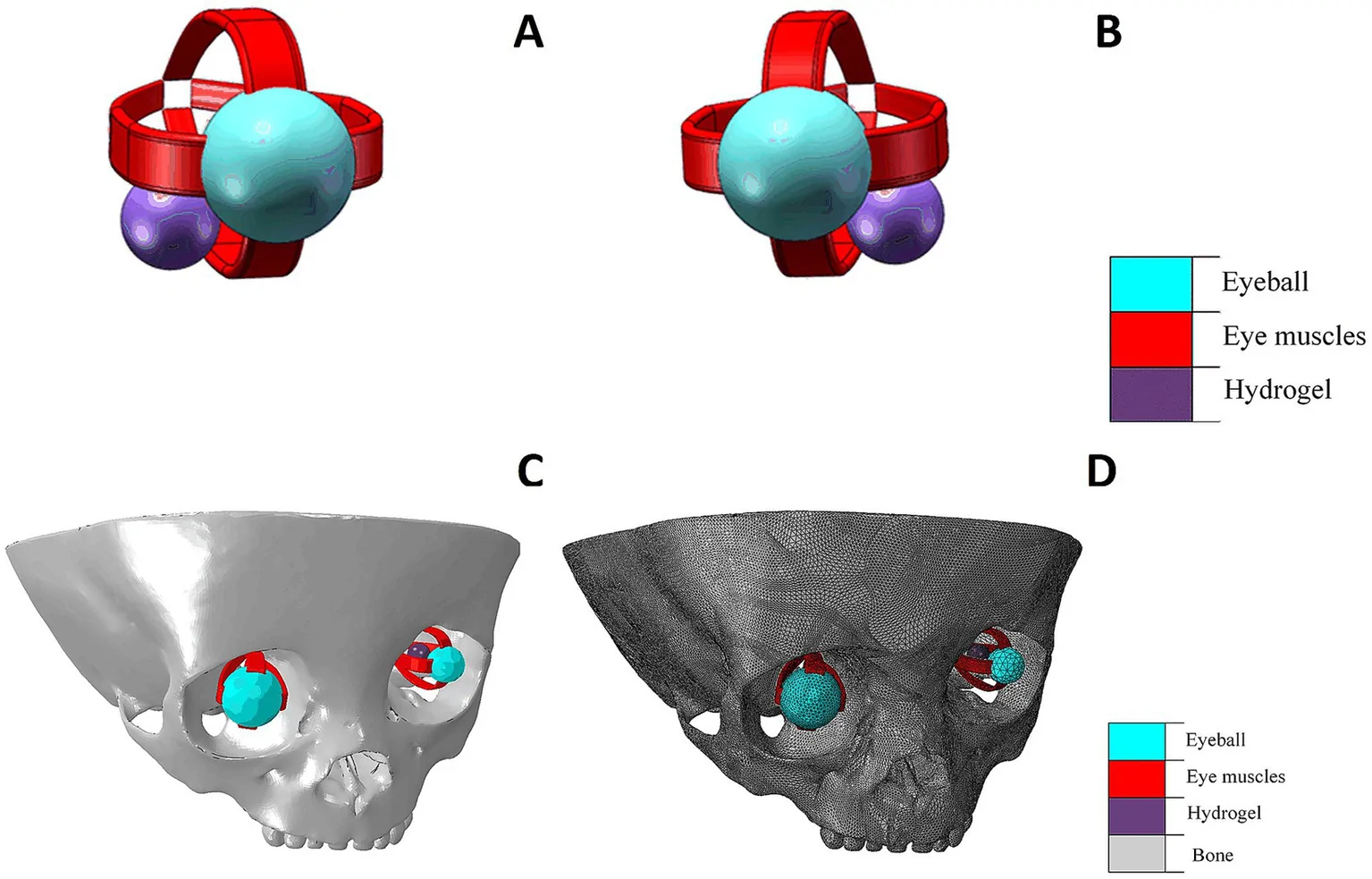
Location of intraorbital hydrogel implant and finite element analysis model. **(A)** The self-inflating spherical hydrogel implant was displaced to the extraconal inferonasal orbital space (Model 1). **(B)** The self-inflating spherical hydrogel implant was displaced to the extraconal inferotemporal orbital space (Model 2). **(C)** Three-dimensional geometrical models of orbit and intraorbital structures. **(D)** Finite element mesh (grid) model used for biomechanical simulation and analysis.

To investigate the biomechanical effects of hydrogel malposition on orbital bone development, a 3 mL spherical hydrogel implant was virtually positioned in two extraconal retrobulbar locations. In Model 1, the implant was placed in the inferonasal region, defined such that the hydrogel surface was tangent to the globe surface, and the vector connecting the centers of the hydrogel and globe formed a 45° angle with the horizontal, vertical, and anteroposterior axes of the globe center. In Model 2, the implant was placed in the inferotemporal region using the same geometric constraints. These two configurations were defined as Model 1 and Model 2 (Figures 1A,B). Residual orbital space between the globe, extraocular muscles, hydrogel, and orbital walls was filled with orbital fat.

Finite element analysis was performed on the reconstructed orbital models (Figures 1C,D), including skull, globe, extraocular muscles, orbital fat, and hydrogel implant. The mesh was generated using linear tetrahedral elements. Mesh density and node numbers are summarized in Table 1. Contact interactions between adjacent structures were defined using frictionless sliding (penalty-based) contact formulation. All materials were assumed to be homogeneous, isotropic, and linearly elastic to simplify numerical simulation. The expanded hydrogel was modeled as a rigid body. Material properties of cranial bone, globe, extraocular muscles, and orbital fat were assigned based on previously published literature (23) (Table 2).

**Table 1:** Number of elements and nodes.

Tissue	Elements	Nodes
Bone	1,653,944	348,397
Eye ball	19,314	28,776
Muscle	39,965	42,353
Fat	2,290,414	3,318,297

**Table 2:** Material properties in the finite element model.

Tissue	Young’s modulus (MPa)	Poisson’s ratio
Bone (23)	11,000	0.326
Eye ball (23)	0.5	0.47
Muscle (23)	11	0.4
Fat (23)	0.047	0.49

In the finite element model, the superior surface of the frontal bone was fully constrained. During simulation, hydrogel expansion was modeled as a volumetric increase up to nine times its initial volume. To realistically simulate the expansion process, a spherical coordinate system was established with the center of the hydrogel as the origin, and radial displacement was applied to the hydrogel surface. Orbital deformation was evaluated at different expansion stages by comparing radial displacement fields.

### The center of four orbital walls

**Figure 2:**
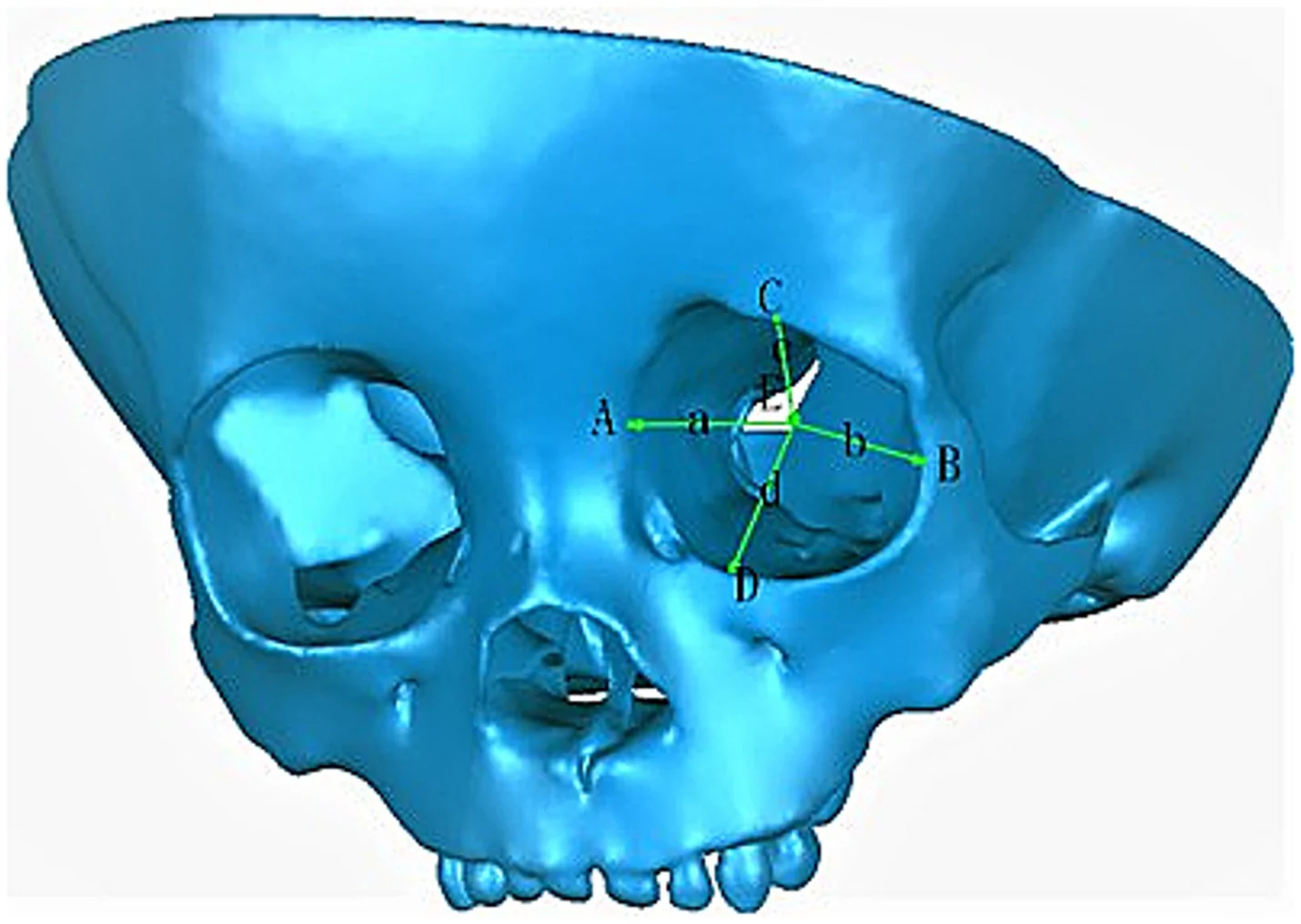
Definition of orbital wall center points in the three-dimensional finite element model. Five anatomical landmark points were identified on the three-dimensional (3D) orbital model, including the ectoconchion **(A)**, dacryon **(B)**, midpoint of the superior orbital rim **(C)**, midpoint of the inferior orbital rim **(D)**, and the lateral margin of the optic canal **(E)**. Based on these reference landmarks, points a, b, c, and d were defined as the geometric center points of the medial, lateral, superior, and inferior orbital walls, respectively. 3D, three-dimensional.

To quantify biomechanical responses, four central points of the orbital walls (a, b, c, and d) were selected for tracking stress and displacement evolution during hydrogel expansion. First, five anatomical landmarks were defined on the orbital model: point A (lacrimal point), point B (lateral orbital rim), point C (midpoint of superior orbital rim), point D (midpoint of inferior orbital rim), and point E (lateral apex of the optic canal). The lateral orbital rim was defined as the intersection between the frontozygomatic suture and the orbital margin, and the lacrimal point was defined as the junction of the maxillary, lacrimal, and frontal bones. A straight line AB was drawn, and its perpendicular bisector intersecting the superior and inferior orbital rims defined points C and D, respectively. Lines AE, BE, CE, and DE were then constructed, and their midpoints were defined as points a, b, c, and d, corresponding to the central regions of the medial, lateral, superior, and inferior orbital walls, respectively (Figure 2).

Although self-inflating hydrogels exhibit swelling behavior and evolving mechanical properties during water absorption, the hydrogel was modeled as a rigid body with prescribed volumetric expansion in the present study to simplify computation and isolate the biomechanical effects of implant position on orbital tissues.

## Results

### The von Mises stress and radial displacement of the orbital walls

**Figure 3:**
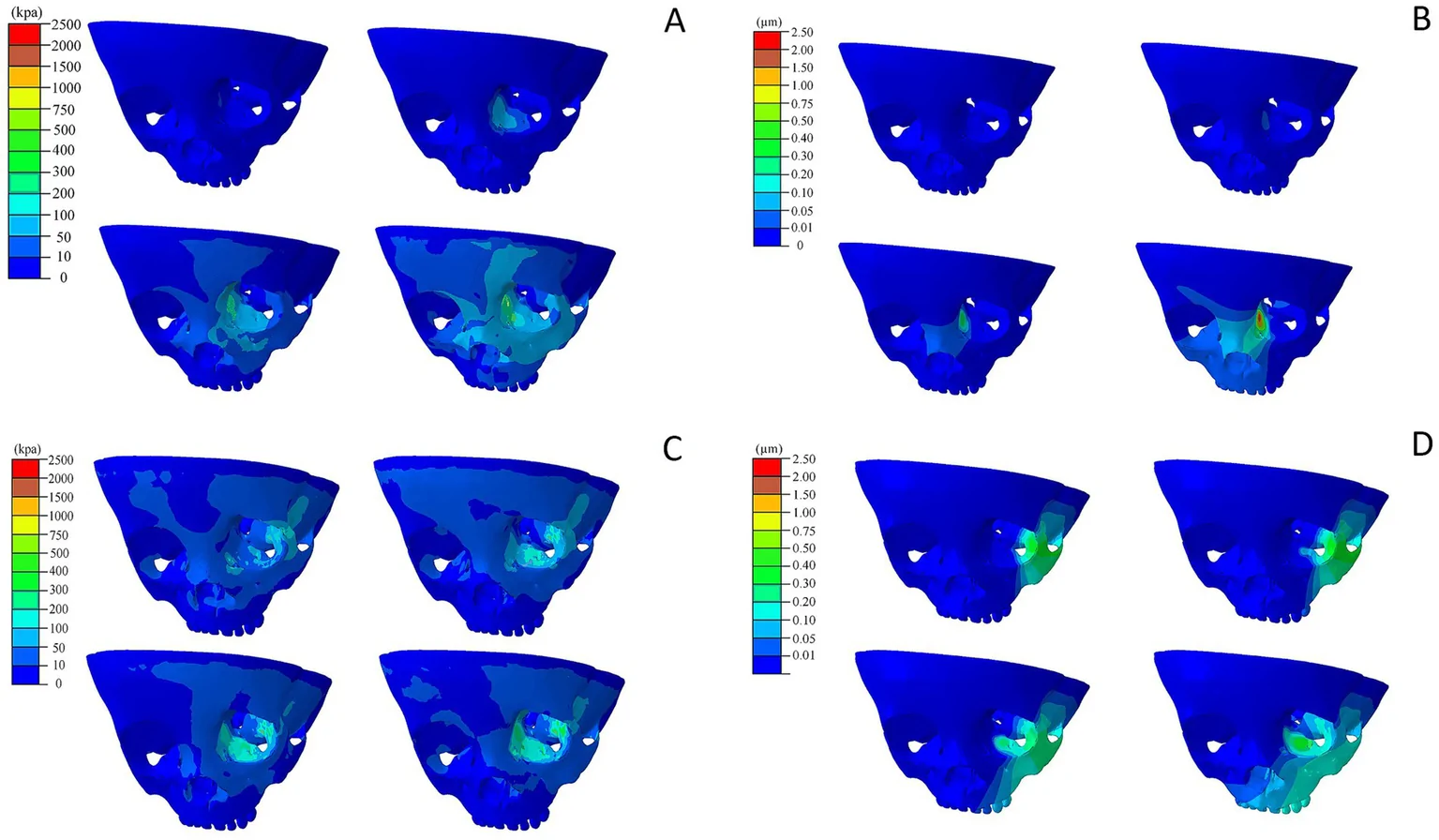
Distribution of von Mises stress and radial displacement of the orbital walls during progressive hydrogel implant expansion. The biomechanical responses of the orbital walls were simulated as the hydrogel implant expanded to 3-, 5-, 7-, and 9-fold of its initial volume. **(A)** Distribution of von Mises stress in Model 1 (extraconal inferonasal displacement). **(B)** Radial displacement distribution in Model 1. **(C)** Distribution of von Mises stress in Model 2 (extraconal inferotemporal displacement). **(D)** Radial displacement distribution in Model 2.

Finite element analysis demonstrated that when the spherical hydrogel implant was positioned in the inferonasal extraconal retrobulbar region (Model 1), the maximum Von Mises stress was primarily concentrated in the inferonasal region of the medial orbital wall. As shown in Figure 3A, when the hydrogel implant expanded to 3-, 5-, 7-, and 9-fold of its original volume, the maximum Von Mises stress reached 10.15 kPa, 50.16 kPa, 200.05 kPa, and 278.15 kPa, respectively. The stress distribution gradually extended from the inferonasal medial orbital wall toward the entire orbit during expansion, while the highest stress remained localized in the inferonasal orbital region, indicating progressive biomechanical loading in this area.

Based on the spherical coordinate system established using the center of the hydrogel sphere as the origin, the regions with maximal radial displacement were generally consistent with those showing maximal equivalent stress (Figure 3B), with the greatest displacement observed in the inferonasal orbital wall. When the self-inflating spherical hydrogel expanded to 3-, 5-, 7-, and 9-fold of its original volume, the maximum orbital bone displacements were 0.045 μm, 0.057 μm, 0.305 μm, and 0.406 μm, respectively.

As shown in Figure 3C, the distribution pattern of maximum Von Mises stress in Model 2 was similar to that observed in Model 1. Initially, stress was concentrated in the orbital wall adjacent to the hydrogel implant, specifically the inferolateral orbital wall, and gradually spread throughout the orbit during hydrogel expansion. Ultimately, the greatest biomechanical stress was observed in the inferotemporal orbital region, while substantial stress was also present on the nasal side of the orbit. When the hydrogel sphere expanded to 3-, 5-, 7-, and 9-fold of its original volume, the maximum Von Mises stresses were 200.05 kPa, 225.15 kPa, 300.24 kPa, and 425.36 kPa, respectively, whereas the maximum orbital bone displacements were 0.201 μm, 0.252 μm, 0.403 μm, and 0.703 μm, respectively.

Under the same scale conditions, the maximum Von Mises stress in the inferior orbital wall of Model 1 was lower than that in the inferolateral orbital wall of Model 2. In terms of radial displacement trends, both models demonstrated greater biomechanical stress and displacement in orbital walls adjacent to the hydrogel implant. However, Model 2 exhibited a more balanced stress distribution across the four orbital walls and more pronounced displacement in the inferotemporal orbital region, suggesting that inferotemporal implant malposition may exert a stronger stimulatory effect on orbital bone development (Figure 3D).

### The von Mises stress and radial displacement of the central orbital walls

**Figure 4:**
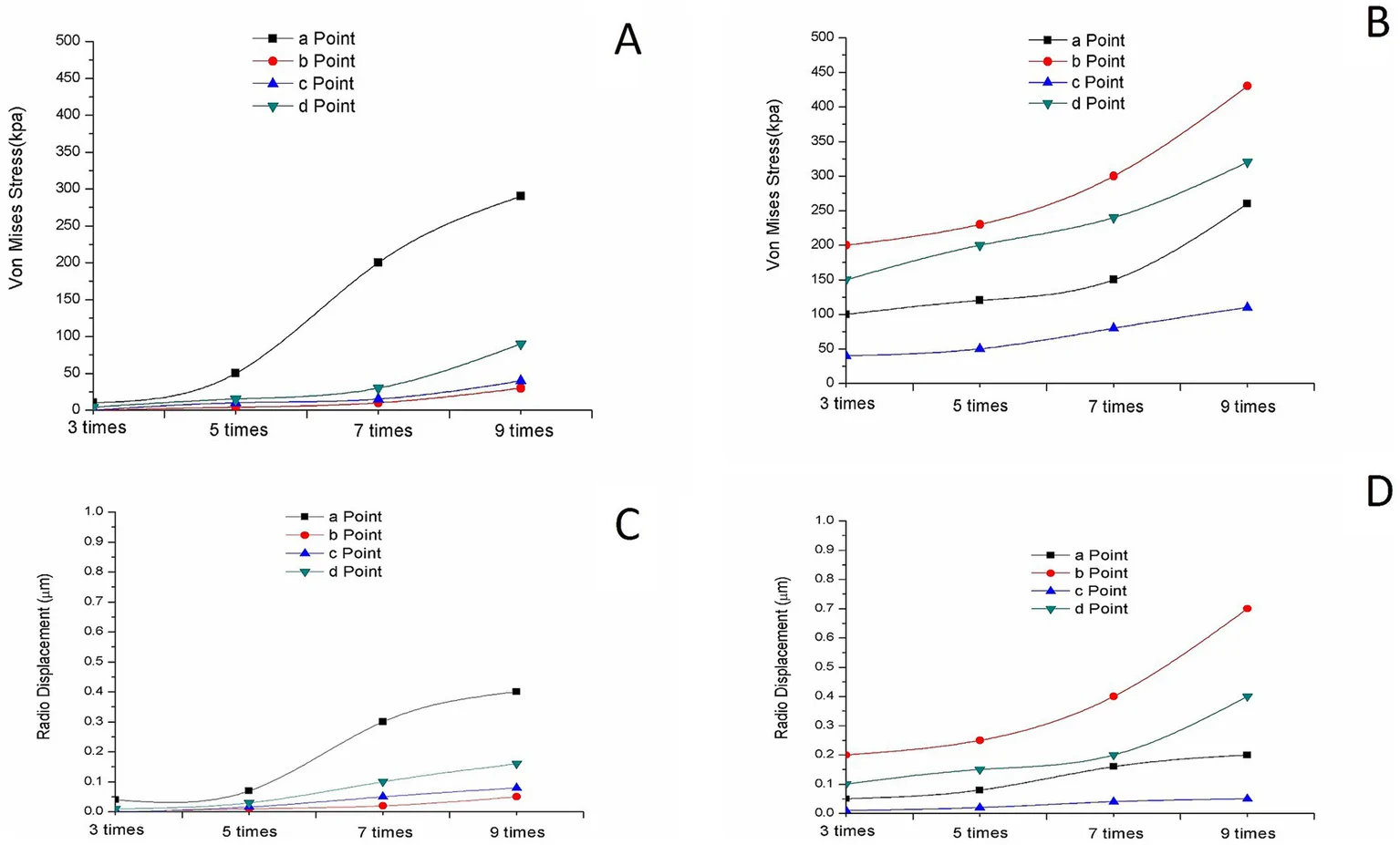
Changes in von Mises stress and radial displacement at the central points of the orbital walls during progressive hydrogel implant expansion. Curve graphs illustrate the biomechanical responses of the central orbital wall points as the hydrogel implant expanded to 3-, 5-, 7-, and 9-fold of its initial volume. **(A)** Von Mises stress at four central orbital wall points in Model 1. **(B)** Von Mises stress at four central orbital wall points in Model 2. **(C)** Radial displacement at four central orbital wall points in Model 1. **(D)** Radial displacement at four central orbital wall points in Model 2.

Analysis of stress and displacement changes at the central points of the four orbital walls (points a, b, c, and d) demonstrated that equivalent stress increased progressively at all points as the hydrogel expanded (Figures 4A,C).

In Model 1, stress at point a was initially minimal but increased substantially with hydrogel expansion, eventually exceeding the values observed at points b, c, and d. The stress magnitude at point d also became greater than those at points b and c, indicating that the maximal stress was consistently concentrated at points a and d (Figure 4A), consistent with the equivalent stress nephograms shown in Figure 3A. Similarly, the displacement curves for Model 1 (Figure 4C) demonstrated that radial displacement at points a and d progressively increased during hydrogel expansion and eventually exceeded that observed at points b and c, corresponding well with the radial displacement maps shown in Figure 4B.

In Model 2, the stresses at points b and d gradually increased with greater amplitude, as shown in Figure 4B. At the final stage of hydrogel expansion, point b exhibited the highest stress value. According to the displacement curves in Figure 4D, radial displacement at point b also became the largest during the later stages of expansion, followed by point d. These findings were consistent with the corresponding equivalent stress and displacement nephograms shown in Figures 3C,D. Compared with Model 1, Model 2 demonstrated more balanced biomechanical stress distribution and greater radial displacement.

Overall, both the biomechanical stress and displacement values of the orbital bones were greater in Model 2 than in Model 1. The trend of stress increase was consistent with the direction of radial displacement. At the final stage of hydrogel expansion, the most pronounced displacement was observed in the inferotemporal orbital region.

Figure 4 illustrates the changes in equivalent stress and radial displacement at the central points of the four orbital walls as the hydrogel sphere expanded to 3-, 5-, 7-, and 9-fold of its initial volume. Panels A and B present the equivalent stress curves for Models 1 and 2, respectively, whereas Panels C and D show the corresponding radial displacement curves.

## Discussion

Microphthalmia is defined as a globe with a total axial length at least two standard deviations below the age-matched mean (9, 11). Severe microphthalmia refers to a corneal diameter <4 mm and axial length <10 mm at birth (11). Congenital microphthalmia is one of the most common hereditary developmental ocular anomalies leading to blindness. It may occur unilaterally or bilaterally, most frequently in a unilateral form, and can be present either in isolation or as part of systemic syndromes (9, 24, 25). The patient included in this study had unilateral severe microphthalmia with a non-functional globe, fulfilling the criteria for severe microphthalmia. The etiology remains unclear but is thought to involve genetic mutations leading to arrested ocular development during embryogenesis (26). Currently, no etiological treatment exists. Orbital hypoplasia is a consistent feature, as orbital growth is strongly influenced by craniofacial bone development, sinus pneumatization (particularly ethmoid sinus), and intraorbital tissue expansion. The presence and growth of the globe are critical for normal orbital development (24, 27). Therefore, early intervention is recommended to stimulate orbital expansion in affected children.

At present, osmotic self-expanding spherical hydrogels are widely used for orbital volume augmentation. These hydrogels, composed of hydrophilic polymers, absorb surrounding fluid and expand to approximately ten times their initial volume. The expansion rate is pre-designed during manufacturing. This technique has been applied clinically in Europe and China with encouraging outcomes.

In our center, more than 60 cases of orbital hydrogel implantation have been performed. However, in 8 cases, postoperative implant displacement occurred, mainly toward the inferonasal or inferotemporal orbit. The biomechanical consequences of such malposition remain unclear, and whether surgical repositioning is necessary has not been established. The hydrogel itself was simplified as a rigid body with prescribed expansion. This assumption neglects the time-dependent swelling process and the progressive changes in material stiffness during water absorption. Although this simplification is suitable for evaluating orbital biomechanical responses, future studies incorporating poroelastic constitutive models or fluid–structure interaction analysis may more accurately simulate hydrogel expansion behavior.

Mechanical loading has long been recognized as one of the principal regulators of bone adaptation (Wolff’s law and mechanostat theory). Increased stress may stimulate osteocytes and osteoblast-mediated remodeling; however, the present computational model only evaluated the mechanical environment rather than directly simulating biological remodeling. Imaging modalities such as ultrasound and CT are commonly used for evaluation, but they cannot capture continuous dynamic expansion, and ultrasound has limited resolution for orbital soft tissues (9). Although CT provides structural detail, radiation exposure remains a concern in pediatric patients (24). Therefore, scans are strictly indicated and performed using pediatric low-dose protocols. Given that implant behavior is fundamentally a biomechanical problem, FEA provides a powerful alternative approach.

Biomechanics applies mechanical principles to biological systems (28). Finite element modeling discretizes complex anatomical structures into small elements for numerical simulation (29), enabling accurate analysis of stress and deformation in irregular geometries (30, 31). It has been widely used in pediatric biomechanics, including child restraint systems, cranial deformation during birth, and pediatric head injury analysis (32–34). The U.S. FDA has also recognized its value in preclinical evaluation (35).

The key to reliable FEA is accurate model construction. Because orbital anatomy varies significantly in congenital microphthalmia, no standardized dataset exists (23, 36, 37). In this study, high-resolution CT data were used to reconstruct orbital and cranial structures using Mimics 17.0, combined with CAD-based modeling of the globe, extraocular muscles, and hydrogel implant. Manual correction was performed to ensure anatomical fidelity.

Material properties were derived from experimental measurements and literature data. Although orbital tissues exhibit nonlinear, anisotropic, viscoelastic behavior, a linear elastic isotropic assumption was adopted to ensure computational stability (38, 39). Two representative malposition sites were defined clinically: inferonasal and inferotemporal regions, located approximately 2 mm posterior to the microphthalmic globe surface. These positions were selected based on surgical observations, as most implants were inserted via conjunctival incisions from either nasal or temporal approaches. Hydrogel expansion was simulated up to ninefold volume increase. Regardless of position, stress initially concentrated in adjacent orbital walls and subsequently propagated throughout the orbit. However, stress magnitude and displacement were significantly greater in the inferotemporal model. Maximum Von Mises stress reached 425.36 kPa in Model 2 versus 278.15 kPa in Model 1, and maximal displacement reached 0.703 μm versus 0.406 μm, respectively.

Further analysis of orbital wall central points (a, b, c, d) confirmed that stress increase preceded displacement, consistent with physiological bone remodeling responses. Notably, inferotemporal loading produced more balanced multi-wall stimulation and greater overall orbital expansion. From a biomechanical perspective, hydrogel-induced stress promotes functional bone adaptation. Regions experiencing higher mechanical loading exhibit enhanced osteogenic activity, leading to orbital enlargement. The inferotemporal region, adjacent to the zygomatic bone, allows greater outward displacement compared with the inferonasal region, which is anatomically constrained by the maxilla. This explains the greater displacement observed in Model 2.

Bone remodeling is regulated by mechanotransduction, whereby osteocytes sense mechanical loading and subsequently regulate osteoblast and osteoclast activity. Moderate mechanical stimulation promotes extracellular matrix deposition and adaptive bone remodeling. Therefore, the stress and displacement distributions observed in the present finite element simulations should be interpreted as biomechanical indicators that may influence orbital bone remodeling rather than direct evidence of bone growth. Consequently, regions exhibiting higher stress and displacement may possess greater osteogenic potential, although biological validation is still required.

Clinically, implant displacement was closely related to surgical approach. Temporal incisions were more frequently associated with inferotemporal malposition, whereas nasal incisions tended to result in inferonasal displacement. Excessive dissection or insufficient soft tissue support may facilitate migration under gravitational and muscular forces. The implant’s smooth surface and hydrophilic expansion behavior further contribute to positional instability, particularly when orbital volume mismatch exists. Importantly, inferotemporal malposition may not always require surgical correction. When the implant remains in the deep orbit without causing superficial deformity, continued observation may be appropriate, as biomechanical stimulation appears sufficient to promote orbital growth. In contrast, superficial displacement with cosmetic deformity warrants repositioning. Finite element analysis provides a quantitative framework for individualized decision-making, enabling prediction of stress distribution based on implant position and orbital anatomy. This may help guide whether observation or surgical intervention is appropriate.

More broadly, recent advances in ophthalmic research have highlighted the increasing role of computational technologies in translating basic research into clinical practice. Quantitative analytical approaches, including biomechanical modeling, electrophysiological assessment, and artificial intelligence, are increasingly being incorporated into individualized ophthalmic diagnosis and treatment planning, providing objective evidence beyond conventional imaging and clinical examination (40). Notably, a recent large-scale study demonstrated that an artificial intelligence framework based on retinal imaging could simultaneously detect multiple ocular and systemic diseases, highlighting the transformative potential of computational models in precision ophthalmology (41). Consistent with this trend, our patient-specific finite element model provides a quantitative biomechanical framework for predicting implant stability and postoperative orbital biomechanics in congenital microphthalmia, thereby supporting personalized surgical planning. Future integration of finite element analysis with multimodal imaging, artificial intelligence, and patient-specific digital modeling may further improve the precision and clinical applicability of orbital reconstruction strategies.

This study has several limitations. First, the finite element model was reconstructed from a single patient and therefore reflects an individualized biomechanical scenario rather than population-level evidence. Second, linear elastic isotropic material properties were assumed for orbital tissues and the hydrogel implant, whereas the true materials exhibit nonlinear and time-dependent behavior. Third, the model predicts only mechanical responses and cannot directly simulate biological bone remodeling or clinical outcomes. Finally, clinical recommendations derived from the present findings should be interpreted cautiously until validated in larger patient cohorts.

## Conclusion

This patient-specific finite element simulation suggests that deep inferotemporal malposition of self-inflating hydrogel implants may still provide favorable biomechanical stimulation for orbital remodeling and therefore may not necessarily require immediate surgical repositioning. However, these findings should be interpreted cautiously because they are based on a single reconstructed case. Further clinical studies and patient-specific finite element analyses are required to validate these preliminary observations. The gradual expansion characteristics of self-inflating hydrogels support their potential feasibility for promoting orbital development, although optimal implant positioning remains important for maximizing therapeutic outcomes.

## Data Availability

The original contributions presented in the study are included in the article/supplementary material, further inquiries can be directed to the corresponding authors.
